# Technological and Therapeutic Advances in Advanced Small Cell Lung Cancer

**DOI:** 10.3390/cancers11101570

**Published:** 2019-10-15

**Authors:** Caroline Lum, Muhammad Alamgeer

**Affiliations:** 1 Department of Medical Oncology, Monash Health and Monash University, Clayton, VIC 3168, Australia; caroline.lum@monashhealth.org; 2 Centre for Cancer Research, Hudson Institute of Medical Research, Monash University, Clayton, VIC 3168, Australia

**Keywords:** small cell lung cancer, targeted therapies, immunotherapies, bio-markers

## Abstract

Small cell lung cancer (SCLC) accounts for approximately 10–15% of all lung cancers. The prognosis is poor with median survival in the advanced stage remaining at around 12 months. Despite applying every known therapeutic approach, no major breakthrough has improved the overall survival in the last 30 years. Historically, experiments performed on conventional cell lines may have limitations of not accurately reflecting the complex biological and genomic heterogeneity of this disease. However, additional knowledge gained from recently developed genetically engineered mouse models (GEMMs) and patient derived xenografts (PDXs) have made encouraging inroads. Whole genome sequencing (WGS) data reveals a high mutational burden and a number of genetic alterations but low frequency of targetable mutations. Despite several failures, considerable therapeutic opportunities have recently emerged. Potentially promising therapies include those targeting DNA damage repair, stem cell/renewal and drug resistant mechanisms. Modest success has also been achieved with immune checkpoint inhibitors while therapeutic exploration of various other components of the immune system is underway. However, the complex heterogeneities reflect the need for accurate bio-markers to translate novel discoveries into clinical benefit. Additionally, the molecular mechanisms that differentiate chemo-sensitive from chemo-refractory disease remain unknown. Obtaining reliable tumour samples by utilising novel techniques such as endobronchial ultrasound guided needle aspiration or adopting to liquid biopsies are becoming popular. This review will focus on recent technological and therapeutic advancements to surmount this recalcitrant disease.

## 1. Introduction

Small cell lung cancer (SCLC) remains a devastating disease, characterised by an aggressive course with early metastasis. Standard first line therapy for extensive stage (ES-SCLC) disease remains platinum-based doublet chemotherapy [1], with response rates (RR) in this setting in the order of 70% [1]. Inevitable progression eventually follows, with a median survival in ES-SCLC remaining at around 12 months [2]. In a minority of the patients, however, relapse occurs within three months; in these cases, the disease is defined as chemorefractory. Although topotecan has been approved as monotherapy for relapsed SCLC, its low RR and short median survival time have been disappointing [3]. In addition, not only have multiple trials of several cytotoxic agents, dose intensification, as well as novel targeted therapies failed to improve the outcomes over the last three decades [4,5], no specific agents to re-sensitise the tumour cells to chemotherapy could be formulated. In response, the focus of drug development has shifted more towards biological or biomarker directed therapies. The major challenge is to develop efficient models to investigate biomarkers in the settings of inter- and intra- tumoural heterogeneity. Current therapeutic focus can be broadly grouped into five major categories: Development and regulatory pathways, DNA damage and repair (DDR), cell cycle inhibitors, epigenetics and immune therapies. This review will focus on these developments as well progress in strategies to distillate effective bio-markers. 

## 2. Genomics and Pathways in SCLC

**Figure 1:**
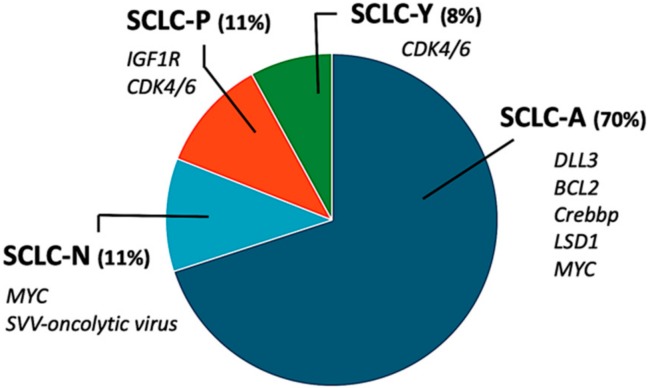
Proposed nomenclature describing small cell lung cancer (SCLC) molecular subtypes. Last letter signifies the most strongly associated transcription factor associated with that particular SCLC subtype. A= (Achaete-scute homologue 1 (ASCL1; also known as ASH1); N= neurogenic differentiation factor 1 (NeuroD1); P = POU class 2 homeobox 3 (POU2F3) and Y= yes-associated protein 1 (YAP1). Presumed potential targets according to subtypes are illustrated in italics below each subtype. (Proportion of each subtype is approximated based on the combined data from multiple studies involving both cell lines and human tissues).

Genomic instability and complex genomic rearrangements are the hallmark of SCLC. High levels of genetic alterations and mutation frequency were found at rates only second to those of melanoma [6,7]. WGS data reveals evidence for ~90% biallelic loss of *TP53* and *RB1* [6,7]. Some tumours with wild type *RB1* have shown to exhibit chromothripsis, where alternative mechanisms of *RB1* deregulation, such as overexpression/amplification of cyclin D1 (CCD1) and/or inactivation of cyclin dependent kinase inhibitor 2A (CDKN2A), have been identified [6,8]. Further comprehensive genomic profiling uncovered alterations at both copy number and mutational levels in several key genes involved in cell cycle regulation (*CDK4/6*), receptor kinase signaling (*KIT, FGFR, IGFR*), transcriptional regulation (*CREBBP, MYCL1, MYCN, MYC),* apoptosis *(SOX2, BCL2*) and Notch signaling/neuroendocrine differentiation (*NOTCH, ASCL1*) [6,9]. Further sequencing on patient samples revealed high mutational burden (a feature associated with exposure to carcinogens in tobacco) [10], but unlike non-small cell lung cancer (NSCLC), potentially targetable oncogenic driver mutations have not so far been tracked down in SCLC [11]. Although ‘classic and variant’ subtypes, based on morphological and biochemical characteristics of SCLC cell lines, were described almost three decades ago [12], it was only in the last five years that a variety of molecular subtypes have emerged [13,14]. Recent profiling studies involving both primary human and mouse tumours supported various models of distinct SCLC molecular subtypes defined by relative expression of key transcription regulators including ASCL1) (achaete-scute homolog 1), NeuroD1 (neurogenic differentiation factor 1), YAP1 (yes-associated protein 1), and POU2F3 (POU domain class 2 homeobox 3) [13]. Subsequently, multiple independent research teams have collaborated together to propose a consistent nomenclature for these subtypes as SCLC-A, SCLC-N, SCLC-Y, and SCLC-P [13] and also hypothesised that these biologically distinct subtypes may also have distinct therapeutic targets (Figure 1). 

The intricate genetic features that differentiate chemosensitive from chemorefractory disease currently remain unknown. Unavailability of longitudinal samples to investigate tumour evolution and mechanisms of acquired chemoresistance has been a major reason for this gap in knowledge. In a gene expression profiling study using SCLC cell lines, McColl et al. showed that the rare *RB1* WT subtype expressed YAPI (currently classified as SCLC-Y) and was chemo-resistant, while INSM1 (insulinoma-associated protein 1) expressing subtype (SCLC-A and SCLC-N) was chemosensitive [14]. Further information was gained from a small study involving serial circulating tumour cells (CTCs), where copy number aberrations (CNAs) data pointed towards distinct profiles in patients with chemorefractory and chemosensitive SCLC [15]. The study also implied that the genetic alterations associated with inherent drug resistance may be different to acquired drug resistance [15]. Larger studies with suitable samples are required to confirm these findings. So far several attempts to target traditional platinum resistance mechanisms have been unsuccessful, while others such as targeting DNA repair system are being explored [16]. 

## 3. SCLC and Genomic Heterogeneity

Genomic heterogeneity is now recognised as a major barrier to the success of conventional therapies in the treatment of cancers [17]. The advent of multi-region sequencing has led to the identification of a previously unknown degree of genomic complexity in solid tumours [18]. However, the landmark findings from analysing multiple tumour sites from a relatively small number of cases, reveal the complexities of genomic evolution and have major implications for the understanding of tumour initiation and progression [19]. Furthermore, they illustrate that analysing multiple tumour sites can reveal the complexities of genomic evolution and heterogeneity that cannot be resolved by single-site sequencing of large cohorts [17]. Though the intratumoural and intertumoural heterogeneity was demonstrated in SCLC cell surface antigens almost three decades ago [20], subclonal architecture of SCLC and its clonal evolution during treatment has not been well delineated, possibly due to lack of multi-site and serial tumour samples. Recent sequencing studies using serial circulating tumour DNA (ctDNA) samples underpinned the genomic landscape of SCLC. A study using serial liquid biopsies from 22 SCLC patients showed post treatment enrichment of mutations associated with chemo-resistance (DNA repair and Notch pathway mutations) using target deep sequencing of 430 cancer genes [21]. The only study to use multi-region sequencing had only one patient with SCLC, revealing that the primary lesion was genomically distinct from the metastatic site and there was heterogeneity in immune related markers [22]. In animal models, Sage et al. showed plasticity in Notch signalling and phenotypic switch from a neuroendocrine to non-neuroendocrine subtype, suggesting the evolution of various subpopulations of SCLC cells and their functional interactions [23]. 

## 4. Targeting Developmental and Regulatory Pathways

### 4.1. Notch Signaling

Notch signaling is involved in cell fate/neuroendocrine differentiation and plays a pivotal role in the development of SCLC [24]. Delta-like ligand 3 (DLL3), an inhibitory ligand for the Notch receptor, has emerged as an attractive therapeutic target in approximately one third of SCLC, where it is highly expressed [25,26]. An antibody drug conjugate (ADC) rovalptizumab tesirine (Rova-T), targeted at DLL3, showed promising pre-clinical and early clinical activity in DLL3 high expressing SCLC [26,27]. In the subsequent phase 2 TRINITY study evaluating Rova-T in patients with DLL3 expressing SCLC who had received at least two prior lines of therapy, overall response rates (ORR) of 16%, with median overall survival (OS) of 5.6 months was reported [28]. Disappointingly, the phase 3 TAHOE study, investigating Rova-T compared to topotecan in the second line was halted early due to poor interim results. Rova-T combination with immune check point inhibitors (ICI) nivolumab and ipilimumab in patients with DLL3 expressing ES-SCLC, in the second line and beyond, showed safety concerns and further optimisation of schedule and dosing was recommended [29]. Disappointingly, the phase 3 MERU study of Rova-T maintenance following first line platinum-based chemotherapy (NCT03033511) was terminated recently due to lack of survival benefit at a pre-planned interim analysis. As a result, further development of this compound was discontinued.

Two novel approaches in targeting DLL3 are Bi-specific T-cell engagers (BiTE®s; AMGEN® Science Thousand Oaks, CA, USA) and chimeric antigen receptor (CAR) T cell therapy. In these forms of immunotherapy, cytotoxic T cells are directed at DLL3 expressing SCLC with the aim of selective destruction [30,31]. A phase 1 trial of AMG757, an anti-DLL3 BiTE® molecule, initially in patients with relapsed SCLC and later in patients with at least SD following first line chemotherapy (NCT03319940) is in progress. AMG119 is an adoptive cellular immunotherapy, consisting of patients’ derived CAR T cells that target DLL3. It has shown potent killing of DLL3 expressing SCLC cells in vitro and is currently undergoing evaluation in a phase 1 trial in SCLC patients who progressed after at least one line of therapy (NCT03392064).

### 4.2. Hedgehog Pathway 

The Hedgehog pathway (HH), plays a key role in stem cell differentiation and neuroendocrine fate and has been found to be activated in SCLC [32,33]. Unlike some other malignancies (basal cell carcinoma, medulloblastoma, and rhabdomyosarcoma), mutations in HH pathway genes are not typically seen in SCLC; however, upregulation of the HH pathway components has been widely reported [32,34]. Preclinical studies have shown that deletion of Smoothened (SMO); a transmembrane protein inhibited in the absence of a HH ligand, leads to inactivation of the HH pathway [35,36]. Subsequently, there has been an interest in the development of SMO inhibitors to target SCLC.

A phase 1 study of sonidegib (LDE225), a selective SMO antagonist, combined with cisplatin and etoposide investigated the safety in ES-SCLC. At the maximum tolerated dose (MTD) of 800 mg, 11 of 15 patients had a partial response (PR), and one patient with *SOX2* amplification continued to have SD after 27 months of maintenance therapy [33]. A similar compound, vismodegib, was studied by Bellani et al. Three treatment arms investigated in 152 patients with newly diagnosed ES-SCLC: Cisplatin and etoposide; cisplatin, etoposide, and vismodegib; cisplatin, etoposide, and cixutumumab (this latter agent targeting insulin like growth factor 1). No statistically significant difference in the progression free survival (PFS), overall survival (OS) or response rate (RR) was observed [35]. Taladegib, also an antagonist of HH/SMO, was evaluated in a phase 1/2 trial in the first line setting of ES-SCLC, in combination with carboplatin and etoposide chemotherapy. The reported ORR was 53.8% [37]. These disappointing results have led to the discontinuation of further clinical development of HH inhibitors in SCLC. 

### 4.3. Receptor Tyrosine Kinase Targeting

Tyrosine kinase inhibitors (TKIs) in general, have failed in SCLC [4,5]. Recently, anlotinib, a multiple receptor tyrosine kinases inhibitor, targeting vascular endothelial growth factor (VEGF) receptor type two and three, the platelet-derived growth factor b (PDGFR-b), and the stem cell-factor receptor (c-Kit) [38], has shown some efficacy. After establishing safety in a phase I study [39], anlotinib was tested in the phase 2 ALTER 1202 trial. Compared to placebo in 120 patients with SCLC who had received at least two prior lines of therapy, anlotinib improved the median PFS (4.1 m versus 0.7 m) and the median OS (7.3 m versus 4.9 m) with improvement in disease control rate (71.6% versus 13.2%). Adverse effects were similar to other TKIs, and included hypertension, anorexia, fatigue, and hand-foot-syndrome [40].

The ALTER0302 phase 2 clinical trial investigated anlotinib in comparison with placebo in 117 patients in the third line treatment for ES-SCLC. Results were also promising, with anlotinib therapy resulting in significantly improved PFS (4.8 m versus 1.2 m) and objective response rate (ORR) (10% versus 0%). There was a non-significant trend towards improvement in median OS (9.3 m versus 6.3 m) [38].

## 5. DNA Damage Response Targeting

DNA damage response (DDR) pathways are intricate and overlapping systems to maintain genomic integrity in the face of DNA damage and stressors [41]. In SCLC, loss of *TP53* and *RB*, together with the activation of oncogenic *MYC* and *SOX2*, results in rapid carcinogenesis where cancer cells become dependent on intact DDR pathways for survival [42,43]. Proteomic and transcriptomic analyses have identified a number of dysregulated DDR pathways such as Poly (ADP-ribose) polymerase (PARP1), checkpoint kinase 1 (CHK1), ataxia telangiectasia mutated (ATM), ataxia telangiectasia and Rad3-related protein (ATR), and WEE1 G2 checkpoint kinase [44,45,46,47,48]. Preclinical evidence also indicates that the compounds targeting these genes might be promising therapeutic options in SCLC. Moreover, targeting DDR machinery is also believed to reverse platinum resistance in this disease [49].

### 5.1. PARP Inhibitors

PARP is a family of nuclear enzymes involved in DDR where it plays a key role in the repair of single strand breaks (SSBs) [42]. Inhibition of PARP results in the conversion of SSBs to double strand breaks (DSBs) and subsequent apoptosis [42]. PARP-1 inhibitors have been successfully utilised in BRCA mutated cancers with homologous repair deficiency (HRD), e.g., ovarian cancer, and in the context of synthetic lethality [50]. In SCLC however, BRCA mutations are not common and PARP inhibition is dependent on a high level of intrinsic stress and genomic instability [44]. Therefore, preclinical studies have shown only limited single-agent cytotoxicity with PARP inhibitors [51] and alternative strategies of combining PARP inhibitors with DNA damaging cytotoxics provided preclinical evidence of potentiation of therapeutic efficacy [44,51,52,53]. Several PARP inhibitors such as, talazoparib, veliparib, niraparib, and olaparib, are being evaluated in combination with chemotherapy and other novel agents for patients with newly diagnosed or relapsed SCLC.

A phase 1 dose escalation study of veliparib in combination with carboplatin and etoposide was conducted in multiple solid tumours, which included 25 patients with ES-SCLC. The combination showed an impressive response rate of 84% but an increase in haematological toxicities, favouring a synergistic effect [54]. A subsequent phase 2 study of this combination in ES-SCLC in the first line setting is ongoing (NCT2289690). In another first line phase II study (ECOG-ACRIN 2511), efficacy of velaprib plus chemotherapy has been disappointing with a modest improvement in PFS (6.1 m versus 5.5 m, HR 0.75, *p* = 0.06) but not OS [55]. 

Talazoparib has been evaluated in a number of BRCA mutant solid malignancies. In SCLC, 23 patients with platinum sensitive disease received single agent talazoparib, and at the established MTD of 1 mg, six patients had a partial response (PR) or stable disease (SD), while the median PFS was 11.1 weeks [56].

PARP inhibitors are also being investigated in the maintenance setting in patients with SCLC, who have not progressed after first line chemotherapy or chemoradiation. The phase 2 STOMP trial, showed no significant benefit of olaparib as a maintenance treatment compared to placebo [57]. In an ongoing phase 3 trial, niraparib is being compared to placebo as maintenance therapy in Chinese patients with ES-SCLC [58]. 

In relapsed ES-SCLC, a phase 2 study investigated temozolomide plus veliparib or placebo. There was a significant improvement in ORR (39% versus 14%, *p* = 0.016) but not in PFS or OS [59]. A subgroup of patients with high schlafen family member 11 (SLFN11) protein expression, had improved survival with the addition of veliparib [59]. Olaparib combined with temozolomide after at least one prior line of therapy showed an early indication of efficacy with 12 out of 29 patients showing a response and a reported median PFS and OS of 87 and 220 days, respectively [60]. Myelosuppression was the most common adverse event. 

PARP inhibitors in combination with other non-cytotoxic therapies are being investigated. In preclinical models, Olaparib in combination with cediranib (VEGF inhibitor) showed evidence for efficacy [61]. In another phase 2 trial, patients with relapsed SCLC received durvalumab in combination with olaparib, but the trial did not meet its pre-set efficacy endpoint of ORR [62]. Ongoing trials of PARP inhibitors in various combinations in SCLC are shown in Table 1. 

### 5.2. CHK and WEE Inhibition

CHK1 along with other proteins such as WEE1 and ATR participates in the regulation of homologous repair and plays a key role in the DNA damage-dependent cell cycle arrest in cells with *TP53* loss [63]. Inhibition of CHK1 appears to have a therapeutic effect in tumours prone to replication stress [63]. In a preclinical study of prexasertib (CHK1 inhibitor), Sen et al. demonstrated its efficacy as monotherapy and improved responses when combined with cisplatin or olaparib. Their efficacy was particularly enhanced in the context of *MYC* amplification or protein overexpression [45]. Prexasertib is being investigated in an ongoing phase 2 trial in patients with ES-SCLC [64].

The DDR protein WEE1 is involved in the inhibition cyclin dependent kinases via phosphorylation and subsequently halts the cell cycle, allowing for DNA repair [65]. Pre-clinical studies have shown efficacy of WEE1 inhibition [66,67]. Of particular interest is a compound AZD1775, which in preclinical SCLC models has demonstrated synergy with olaparib. The combination was found to be more active than cisplatin and etoposide chemotherapy [68]. AZD1775 has been investigated in a phase 1 study in combination with chemotherapy (cisplatin, carboplatin, or gemcitabine) in patients with refractory solid tumours and found to be safe and tolerable [69]. Of 176 evaluable patients, 10% had a PR and 53% SD, and the RR was favourable in patients with *TP53* mutation compared with the wild type (21% versus 12%) [69]. AZD1775 is currently being evaluated in two ongoing phase 2 trials in relapsed SCLC (NCT02593019, NCT02688907).

## 6. Epigenetic Targeting

### 6.1. Aurora Kinase Inhibition

Aurora kinases A and B (AURK A/B) are serine/threonine kinases that play key roles in the regulation of mitosis. In the absence of p53, AURK A/B provide growth advantage, particularly in *MYC* altered SCLC [70]. Preclinical studies have demonstrated activity of AURK A/B inhibitors in SCLC [71,72].

Alisertib, an inhibitor of AURK A, was investigated in a phase 1/2 trial of multiple tumour types including SCLC [73]. In the phase 2 part, alisertib as a monotherapy showed a PR in 10 of 48 patients with SCLC [74]. In another study, alisertib in combination with paclitaxel in relapsed SCLC, showed improvement in PFS (101 days) compared to 66 days with placebo and also a trend towards improvement in OS [75]. This agent is being investigated further in SCLC. LY3295668 Erbumine is another AURK A inhibitor being tested in ES-SCLC (NCT03898791). Chiauranib, a multiple kinase inhibitor including AURK B, is currently being investigated in a phase 1 trial in patients with SCLC (NCT03216343).

### 6.2. Lurbinectedin

Lurbinectedin is a novel and selective inhibitor of oncogenic transcription. By inhibiting trans-activated RNA polymerase II transcription, it causes double stranded DNA breaks leading to apoptosis [76]. Further, by inhibiting transcription in the tumour associated macrophages, lurbinectedin downregulates immune-suppressive cytokines such as interleukin-6 (IL-6), interleukin-8 (IL-8), C-C motif chemokine 2 (CCL2) and VEGF [77]. A phase 1 study of lurbinectedin in combination with doxorubicin showed promising activity with a complete response (CR) rate of 8% and PR rate of 50% [76]. Outcomes were more favourable in patients with platinum-sensitive disease than refractory, with a reported median PFS of 5.8 months compared with 3.5 months, respectively. The main grade three or more adverse effects included myelosuppression (22.9%) and fatigue (6.7%) [76].

Efficacy as monotherapy has been demonstrated in two phase 2 studies. A study by Paz-Ares et al. included 105 patients with ES-SCLC, in which the ORR was 35.2%. Outcomes were more favourable in patients with sensitive disease, with median OS of 11.9 months compared to only 5.0 months in patients with refractory disease [78]. Another basket study by Perez et al. showed an ORR of 39.3% with overall clinical benefit rate of 50.8% in 61 evaluable patients with recurrent ES-SCLC. The reported median PFS was 4.2 months. [79] 

With promising early phase results, lurbinectedin has received orphan drug designation by the United States Food and Drug Administration (FDA) in 2019. An ongoing randomised phase III ATLANTIS trial of lurbinectedin plus doxorubicin compared to investigator’s choice chemotherapy CAV (cyclophosphamide, doxorubicin, vincristine) or topotecan has completed accrual and should provide additional evidence of the efficacy in SCLC [80].

### 6.3. Carfilzomib

Carfilzomib is a selective proteasome inhibitor being investigated in a number of malignancies including SCLC. The safety of carfilzomib in combination with irinotecan was established in a phase 1 trial [81]. This combination was further evaluated in a phase 2 trial of 62 relapsed SCLC patients. The median PFS of 3.6 months and median OS of 6.9 months was observed and the results were similar for patients with both platinum sensitive and refractory disease [82]. 

In another Phase 1b study in previously untreated ES-SCLC, carfilzomib was administered with carboplatin and etoposide and continued as monotherapy in patients with at least stable disease. The combination was quite toxic with 75% of patients having grade three or four adverse effects. Preliminary efficacy results included ORR of 16/30 (53.3%) and median PFS of 4.4 months (95% CI 3.2–5.8) [83].

Other dysregulated epigenetic processes, such as gene promoter methylation and histone acetylation, have also been targeted in SCLC. Vorinostat and belinostat, the two histone deacetylase (HDAC) inhibitors, have synergistic activity when added to chemotherapy [84,85]. Clinical trials investigating the combination of vorinostat (NCT00702962) and belinostat (NCT00926640) with platinum and etoposide in the first line treatment of patients with ES-SCLC are in progress.

## 7. Cell Cycle Targeting

### CDK4/6 Inhibitors

Cyclin dependent kinases (CDK) 4/6 promote the proliferation of different cell types including haematopoietic stem progenitor cells (HSPCs) by phosphorylating retinoblastoma protein (Rb) [86].

CDK4/6 inhibitors are being investigated and used in a number of malignancies, and have proven efficacy in breast cancer [87]. In RB deficient SCLC mouse models, CDK4/6 inhibitors have shown a myeloprotective and synergistic anti-cancer effect when combined with cisplatin. In preclinical mouse models, G1T28 induced CDK4/6 inhibition not only led to the protection of immune cells from cytotoxic effects of cisplatin but also resulted in tumour volume reduction [86]. Further trials of G1T28 in combination with chemotherapy in first (NCT02499770) and second line are ongoing (NCT02514447). 

Trilaciclib, another CDK4/6 inhibitor was investigated in a phase 2 trial of 91 previously treated ES-SCLC. Treatment with trilaciclib and topotecan resulted in a significant reduction in occurrence and duration of severe neutropenia, compared with placebo (40.6% versus 75.6% and two days versus eight days, respectively), as well as a reduced need for blood transfusions, growth factor support, and dose reductions [88]. Larger studies are needed to determine if CDK4/6 inhibitors will have a clinically meaningful place in the management of SCLC. However, CDK4/6 inhibitors act primarily by blocking RB phosphorylation and hence inducing G1 cell cycle arrest [89]. The tumour cells with RB loss by definition may be resistant to these agents, presumably due to lack the canonical target of these agents. Therefore, the rare RB1 WT SCLC (~10%) should be more sensitive to CDK4/6 inhibitors. A clinical trial in this setting has already been planned (NCT 04010357).

## 8. Targeting Immune Mechanisms

Since SCLC exhibit characteristic features of response to immunotherapies (high somatic mutational burden, genomic instability, and association with paraneoplastic syndromes) [7,90], a stronger rationale to target this mechanism had emerged. Thus, several trials including immune checkpoints inhibitors (CPIs), tumour vaccine, antigenic targets, and adoptive cellular immunotherapy in SCLC were initiated. However, the results in SCLC have been somewhat disappointing so far and certainly inferior to what has been achieved in other cancer types such as renal cell carcinoma, melanoma, and non-small cell lung cancer. To date, the largest data available is with CPIs that target cytotoxic T-lymphocyte-associated protein 4 (CTLA-4) and programmed death protein 1 (PD-1), summarised as follows.

A phase 3 trial by Reck et al. investigated chemotherapy (platinum agent plus etoposide) plus ipilimumab, a CTLA-4 antibody, versus chemotherapy plus placebo in the first line setting of ES-SCLC. The dosing of ipilimumab was 10 mg/kg every three weeks for four doses followed by a maintenance dose of ipilimumab every 12 weeks. Results were disappointing with no clinically significant difference in PFS or OS [91]. Similarly, treatment with single agent anti-PD-1 antibody, nivolumab, did not improve response rates or survival over standard chemotherapy in patients with relapsed SCLC [92]. Subsequent to the observation of relative lack of efficacy, it was postulated that low expression of programmed death-ligand 1 (PD-L1) and an overall immunosuppressive environment characterised by the downregulation of major histocompatibility complex (MHC) class 1, poor T cell infiltration, and presence of myeloid-derived suppressor cells in SCLC, single agent check-point blockade may not be efficacious on its own and combination strategies thereafter have emerged.

The phase 1/2 CHECKMATE-032 trial included a patient cohort with ES-SCLC having progressed after at least one prior line of therapy. This trial investigated nivolumab monotherapy compared to nivolumab plus ipilimumab at two different doses, with ORR as the primary endpoint [93]. In an updated analysis of this cohort, the ORR was 11% in the nivolumab monotherapy arm, and 23% in the nivolumab 1 mg/kg and ipilimumab 3 mg/kg arm. Median PFS was 1.4 months in the nivolumab monotherapy and nivolumab 3 mg/kg and ipilimumab 1 mg/kg arms, and 2.6 months in the nivolumab 1 mg/kg and ipilimumab 3 mg/kg arms, while the median OS was 4.4 and 7.7 months, respectively [94]. Responses occurred regardless of the PDL1 expression. In an exploratory analysis, the ORR was significantly higher with nivolumab plus ipilimumab in patients with a high tumour mutational burden (TMB) (46%) compared with medium (16%) and low TMB (22%), respectively. Based on these results, nivolumab was FDA approved for SCLC as a third line treatment in August 2018.

Nivolumab and ipilimumab (1 mg/kg and 3 mg/kg, respectively) followed by maintenance nivolumab (240 mg flat dose) is being investigated in the phase 2, second line BIOLUMA trial. Interim data from the all-comer cohort (N = 18) has shown an ORR of 38.8% and a disease control rate of 55.5% [95]. The combination was quite toxic with two patients dying of immune related adverse effects (pneumonitis and encephalitis). This study later amended the protocol to include only patients with high TMB to ensure balance of treatment benefits and risks. 

Based on encouraging results from the Checkmate-032 trial, the CheckMate-451 phase 3 trial in 834 patients with ES-SCLC investigating maintenance nivolumab or nivolumab with ipilimumab following the first line platinum doublet was initiated. Unfortunately, this trial did not meet its primary endpoint of OS [96]. 

Another anti-PD1 agent pembrolizumab has been investigated in the phase 1b Keynote 028 and phase 2 Keynote 158. Both studies included patients with previously treated advanced SCLC. In Keynote 028 patients were required to be programmed death-ligand 1 (PDL1) positive (defined as ≥1%), but this was not required in Keynote 158. The primary outcome ORR was 33.3% and 18.7%, respectively [97,98]. In a pooled analysis of these two trials, involving 83 patients ORR was 19.3% (95% CI, 11.4–29.4), with 2 CRs and 14 PRs. Median PFS was two months (95% CI, 1.9–3.4) and median OS 7.7 months (95% CI, 5.2–10.1). In June 2019, the FDA approved pembrolizumab for metastatic SCLC in patients who failed at least two prior lines of treatment. 

Due to the aggressive nature of this disease, only a minority of patients will be expected to survive and hence benefit from third line immunotherapy treatment. A number of early phase trials have therefore been performed. The most promising results thus far have been with atezolizumab (anti PD-L1 antibody). The phase 3 IMpower133 trial evaluated chemotherapy (platinum agent plus etoposide) with atezolizumab or placebo in patients with ES-SCLC in the first line setting. The atezolizumab/placebo were continued as maintenance therapy in responders or those with SD. There was a significant improvement in the median OS in the atezolizumab group (12.3 m versus 10.3 m) and the median PFS (5.2 m versus 4.3 m) [99]. Based on these results the FDA has recently approved atezolizumab as a first line treatment for ES-SCLC in combination with chemotherapy. Almost similar results were produced by the phase III CASPIAN trial when durvalumab was added to the chemotherapy in first line treatment of ES-SCLC [100] (Table 2). 

Table 2 summarises the completed and ongoing clinical trials of immune check point inhibitors in SCLC.

Several tumour vaccines demonstrated limited efficacy when investigated in SCLC. Direct antigen targeting of ganglioside antigen (GD3) with BEC2/bacilli Calmette-Guerin (BCG) showed promising preclinical and early clinical activity [105] but failed in a phase II trial [106]. More recently tumour antigen ganglioside fucosyl-GM1 (FucGM1) targeting with BMS-986012, a novel, nonfucosylated, IgG antibody, showed promising preclinical activity [107]. Further phase I studies in treatment naïve as well as in relapsed/refractory SCLC are ongoing (NCT02815592, NCT02247349). There has not been much success with anti-cancer vaccines. As an example, INGN-225, a dendritic cell based p53 vaccine, did not improve survival, despite improving immune response with enhanced chemotherapy effect in preclinical models [108,109]. Interferons (IFN) were amongst the first immunotherapy cytokines evaluated in SCLC showing survival benefit in a phase II trial [110] but also failed in phase III [111]. 

## 9. Advances in Biomarkers

To date there has been limited incorporation of informative biomarkers in SCLC clinical trials due in part to lack of quality tumour samples and the absence of clinically relevant biomarkers for predicting efficacy of cytotoxic chemotherapy. More concerningly, most of the available tumour samples were obtained at a single timepoint in a treatment naïve baseline scenario, that gave limited insight into the complexities of tumour heterogeneity, evolution, and drug resistance mechanisms. Such biomarker studies require tumour samples from the chemoresistant or chemorefractory patients, which are not always available, thus leading to a heavy reliance on conventional cell lines that may not accurately reflect the complex biological and genomic heterogeneity of the human disease [112]. As a result, efforts to explore readily accessible surrogates for tumour tissue in SCLC have expanded. Multiple studies have demonstrated the utility of circulating bio-markers, that may serve as important and renewable recourses to study resistant mechanisms and develop precision medicine [113]. 

### 9.1. Progress in Liquid Biopsies

Plasma circulating tumour cells (CTCs) and circulating cell free DNA (cfDNA) are emerging as potentially useful rapid tools in SCLC and other malignancies. Such ‘liquid biopsies’ have potential utility in serving as a rapid non-invasive diagnostic tool including identification of mutations, and as predictive and prognostic biomarkers. With potential scope for mutational analysis and serial testing over time to monitor patients’ responses to therapies and disease recurrence or progression, liquid biopsies have been deemed an attractive recent technological development. 

In SCLC, CTCs have been implicated in metastatic process and found to correlate with disease extent [114]. Studies have demonstrated that baseline CTC count is prognostic, and reduction in the CTC count following initial cycles of chemotherapy correlate with patient outcomes [114,115,116,117]. In a correlative study performed by Bellani et al. (investigating chemotherapy, vismodegib, and cixutumumab in newly diagnosed ES-SCLC), significant results in efficacy were only found in an exploratory analysis looking at patients with low CTC counts at baseline compared to those with high baseline CTC counts [35]. Further, Messaritakis et al. derived blood samples from 66 patients with SCLC, and found CTC level to reduce significantly from baseline after the commencement of treatment, followed by a significant increase at relapse [118]. Other studies however have failed to show such a correlation [119]. While several technologies have been utilised to explore the clinical utility of CTCs, technical differences such as throughput, specificity, false-negative/false positive rates, and cost issues need to be overcome for uniformity in results and thus widespread application. 

With advances in next-generation sequencing (NGS) and single-cell sequencing (SCS) technologies, scientists can obtain the complete genome of a CTC and compare it with corresponding primary and metastatic tumours [113,120]. Molecular analysis of CTCs has predicted chemosensitive or refractory disease and subsequent patient outcomes [121]. With specific focus on copy number aberrations (CNAs), Su et al. showed utility of single cell sequencing of CTC with regards to survival, chemotherapy response, and heterogeneity by tracing allele specific CNAs in CTCs isolated at different timepoints of chemotherapy [120]. Phenotypic heterogeneity was also found with regards to expression of Bcl-2, CK, Vim, and M30 [120]. Implantation of CTCs into immunodeficient mice to generate CTC-derived explants may become a useful alternative to patient derived xenograft (PDX) models to effectively mirror the donor tumour. Further developments in this area are ongoing.

A number of studies have shown a promising viability of cell free DNA (cfDNA), as an alternative to tissue genotyping [122,123,124]. In a study of 27 patients with SCLC, Almodovar et al. used cfDNA assay to detect somatic variants (including *TP53, RB1, NOTCH, KIT, PTEN,* and *MYC*). They were also able to track tumour recurrence before radiographic evidence of progression by longitudinal tracking of mutational burden in cfDNA [125]. In the IMpower 133 trial, blood-based analysis of tumour mutational burden yielded high quality data for analysis, but no difference between atezolizumab and placebo was found. Few other clinical studies have confirmed the potential utility of cfDNA. Further, bio-marker data from the Keynote-604 and the CASPIAN trials are awaited. 

### 9.2. Potential Biomarkers 

SFLN11, implicated in DNA damage repair deficiency is an emerging marker of great interest. Using patient derived xenograft models, Gardner et al. demonstrated that suppression of SFLN11 is associated with acquired chemoresistance in SCLC [126]. However, SLFN11 as a predictive biomarker for response to PARP inhibitors has been more promising. In a preclinical study, integrated proteomic, transcriptomic, and genomic analyses showed that high SLFN11 predicted response to PARP inhibition, whereas DDR and HRD did not [52]. In the phase 2 trial of veliparib with temozolomide, SLFN11 positive tumours had improved survival, while PARP-1 expression did not predict clinical outcome [59]. In another study, high levels of DNA repair proteins were predictive, and that activation of the PI3K pathway was associated with greater resistance to PARP inhibition [53].

Tumour mutational burden (TMB) has emerged as a possible key predictor of the response to immunotherapeutic agents in SCLC. In the Checkmate-032 study, employing whole exome sequencing (WES), high TMB (defined as ≥248 mutations) had superior outcomes in comparison to patients with lower TMB, reporting one year OS rates of 35% with nivolumab monotherapy and 62% for ipilimumab and nivolumab therapy [127]. In a retrospective analysis by et al., targeted NGS evaluated TMB in SCLC as a predictive biomarker of response to immunotherapy. They also found high TMB correlated with superior outcomes with immunotherapy, but not with chemotherapy [128]. Conversely, IMpower133 investigating atezolizumab in SCLC did not find a clear association between blood based TMB and outcomes [99]. In a study by Gu et al. a positive correlation between TMB and KMT2C mutation may suggest a possible surrogate marker, but clinical confirmation is required [129]. Further data on clinical utility of TMB in SCLC is awaited. 

The ongoing BIOLUMA trial evaluates efficacy and safety of nivolumab and ipilimumab in lung cancer with a broad translational program to identify potential biomarkers predictive of response and/or resistance including WES of serial biopsies, functional analysis of peripheral T-cells, and gut microbiome analyses [95]. 

Unlike NSCLC, PDL1 expression in SCLC is less common and not proven to be predictive. In a study by Carvajal-Hausdorf et al. involving 90 SCLC samples, only 7.3% expressed PDL1; however, they found B7-H3 being expressed in almost 65% and may be associated with immune invasion in SCLC [130]. 

Multiple other biomarkers have been identified in SCLC and their potential in clinical practice remains an active area of research. Examples include *MYC* amplification and response to aurora kinase inhibitors [131], MicroRNAs and chemoresistance [132] and CD47 expression and immune escape [133]. Given phenotypic and genomic heterogeneity of the disease, a single biomarker may not be sufficient to predict the response to therapy. 

## 10. Conclusions

Novel enabling technologies and tools for basic and clinical research aligned with collaborative focus on experimental and analytical capabilities addressing heterogeneity and complexity of biology in SCLC, have greatly improved our understanding of the disease. Concerted efforts are underway to underpin the genomics, molecular profiling, resistance mechanisms, and novel therapies in SCLC. Considerable progress in understanding the molecular biology has been made recently. In particular, the converging genomic data from human and mouse tissue samples leading to an evolving classification of SCLC into molecular subtypes is being anticipated as a major development. Based on these emerging genomic data and a focus on several potential targets, clinical trials are carefully being planned. To date, therapeutic targeting of DNA damage repair and immune mechanisms have yielded encouraging signals. However, extreme plasticity of SCLC due to presence and evolution of various subpopulations and their functional interactions is posing a high degree of hindrance. More refined and diversified therapeutic strategies connected with basic research to bridge the gaps in the knowledge of the disease as well as identification of clinically meaningful biomarkers will be needed. To achieve these goals, accurate preclinical models and high-quality tissue samples are essential.

## Figures and Tables

**Table 1:** Ongoing clinical trials of Poly (ADP-ribose) polymerase (PARP)inhibitors in small cell lung cancer (SCLC).

Indication	Phase	Treatment/Intervention	Primary Outcome	ClinicalTrials.gov Identifier
ES-SCLC following response to first line platinum-based chemotherapy	III	Niraparib *Vs*. Placebo	PFS OS	NCT03516084
ES-SCLC following response to first line platinum-based chemotherapy	Ib/II	Niraparib plus temozolomide *Vs.* Best supportive care	RP2D PFS	NCT03830918
Multiple tumours including Relapsed/refractory SCLC	I/II	CRLX101 plus olaparib	RP2D/MTD PFS (Expansion)	NCT02769962
ES-SCLC, following response to first line platinum-based chemotherapy	II	Rucaparib plus nivolumab	PFS	NCT03958045
Multiple tumours including relapsed SCLC	I/II	Olaparib plus MEDI4736 *Vs.* Olaparib plus MEDI4736 plus bevacizumab	DCR Safety and tolerability ORR	NCT02734004
Multiple advanced tumours including LS- or ES-SCLC At least one prior line platinum-based chemotherapy	II	Cediranib plus olaparib	ORR	NCT02498613

DCR: Disease control rate (CR + PR + SD); ES-SCLC: Extensive stage small cell lung cancer; LS-SCLC: Limited stage small cell lung cancer; MTD: Maximum tolerated dose; ORR: Objective response rate; OS: Overall survival; PFS: Progression free survival; RP2D: Recommended phase 2 dose.

**Table 2:** Clinical trials involving immune checkpoint inhibitors in SCLC.

Trial	Phase	Patients (n)	Treatments	ORR (%)	mPFS (Months)	mOS (Months)
**First line**						
NCT01331525 [101]	II	42	Ipilimumab + carboplatin + etoposide	72.4	6.9	17.0
CA184-156 [91]	III	954	Ipilimumab + platinum + etoposide Vs Placebo + platinum + etoposide	62 62	4.6 4.4 HR 0.85, *p* = 0.016	11 10.9 HR 0.94 *p* = 0.38
Impower-133 [99]	III	403	Atezolizumab + carboplatin + etoposide Vs Placebo + carboplatin + etoposide	60.2 64.4	5.2 4.3 HR 0.77, *p* = 0.02	12.3 10.3 HR 0.70 *p* = 0.007
Keynote-604	III	453	Pembrolizumab + Platinum/Etoposide	Ongoing		
CASPIAN [100]	III	988	Durvalumab + Tremelimumab + Platinum/Etoposide Vs Durvalumab + Platinum/Etoposide Vs Platinum/Etoposide	67.9 57.6	5.1 5.4 HR 0.78	13.0 10.3 HR 0.73 *p* = 0.005
**Maintenance after first line chemotherapy**						
NCT02359019 [102]	II	45	Pembrolizumab	11.1	1.4	9.6
CheckMate-451 [96]	III	834	Nivolumab 1 mg/kg + ipilimumab 3 mg/kg Vs Nivolumab 240 mg every 2 week Vs Placebo	NA	NA	9.2 9.6 HR 0.92 *p* = 0.37
**Second line and beyond**						
CheckMate-032 [93]	I/II	213	Nivolumab 3 mg/kg Vs Nivolumab 1 mg/kg + ipilimumab 3 mg/kg Vs Nivolumab 3 mg/kg + ipilimumab 1 mg/kg	11 23 18	1.4 2.8 1.4	4.4 7.7 6.0
KEYNOTE-028 [97]	Ib	24	Pembrolizumab	33.3	1.9	9.7
KEYNOTE-158 [98]	II	107	Pembrolizumab	18.7	2.0	9.1
IFCT-1603 [103]	II	73	Atezolizumab Vs Chemotherapy (topotecan/re-induction)	2.3 10	1.4 4.3	9.5 8.7 HR 0.84 *p* = 0.60
CheckMate-331 [92]	III	569	Nivolumab Vs Topotecan or amrubicin	39 47	1.5 3.8 HR 1.41	7.5 8.4 HR 0.86
NCT02261220 [104]	I	30	Durvalumab + tremelimumab	13.3	1.8	7.9
NCT03083691 BIOLUMA [95]	II	106	Nivolumab 1 mg/kg + Ipilimumab 3 mg/kg x 4 followed by nivolumab flat dose 240 mg maintenance	38.8 *	NA	NA

*All-comer cohort N = 18, trial is ongoing with data on high tumour mutational burden (TMB) patients pending. HR: hazard ratio; mOS: median overall survival; mPFS: median progression free survival; NA: not available; ORR: objective response rate; SCLC: small cell lung cancer.
